# Neck muscle afferents influence oromotor and cardiorespiratory brainstem neural circuits

**DOI:** 10.1007/s00429-014-0734-8

**Published:** 2014-03-05

**Authors:** I. J. Edwards, V. K. Lall, J. F. Paton, Y. Yanagawa, G. Szabo, S. A. Deuchars, J. Deuchars

**Affiliations:** 1 School of Biomedical Sciences, University of Leeds, Leeds, LS2 9JT UK; 2 School of Physiology and Pharmacology, Bristol Heart Institute, University of Bristol, Medical Sciences Building, Bristol, BS8 1TD UK; 3 Department of Genetic and Behavioral Neuroscience, Gunma University Graduate School of Medicine JST, CREST, Maebashi, 371-8511 Japan; 4 Department of Gene Technology and Developmental Neurobiology, Institute of Experimental Medicine, Budapest, 1450 Hungary

**Keywords:** Proprioception, Autonomic, Immunohistochemistry, Electrophysiology

## Abstract

Sensory information arising from the upper neck is important in the reflex control of posture and eye position. It has also been linked to the autonomic control of the cardiovascular and respiratory systems. Whiplash associated disorders (WAD) and cervical dystonia, which involve disturbance to the neck region, can often present with abnormalities to the oromotor, respiratory and cardiovascular systems. We investigated the potential neural pathways underlying such symptoms. Simulating neck afferent activity by electrical stimulation of the second cervical nerve in a working heart brainstem preparation (WHBP) altered the pattern of central respiratory drive and increased perfusion pressure. Tracing central targets of these sensory afferents revealed projections to the intermedius nucleus of the medulla (InM). These anterogradely labelled afferents co-localised with parvalbumin and vesicular glutamate transporter 1 indicating that they are proprioceptive. Anterograde tracing from the InM identified projections to brain regions involved in respiratory, cardiovascular, postural and oro-facial behaviours—the neighbouring hypoglossal nucleus, facial and motor trigeminal nuclei, parabrachial nuclei, rostral and caudal ventrolateral medulla and nucleus ambiguus. In brain slices, electrical stimulation of afferent fibre tracts lateral to the cuneate nucleus monosynaptically excited InM neurones. Direct stimulation of the InM in the WHBP mimicked the response of second cervical nerve stimulation. These results provide evidence of pathways linking upper cervical sensory afferents with CNS areas involved in autonomic and oromotor control, via the InM. Disruption of these neuronal pathways could, therefore, explain the dysphagic and cardiorespiratory abnormalities which may accompany cervical dystonia and WAD.

## Introduction

The intermedius nucleus of the medulla (InM) is a neurochemically diverse perihypoglossal nucleus (Edwards et al. 2007, 2009) with no known function. Furthermore, very little is known regarding the anatomical connectivity of the nucleus. We have previously identified a monosynaptic projection from the InM into the neighbouring nucleus of the solitary tract (NTS) using electrophysiology (Edwards et al. 2007), indicating a possible role in autonomic and/or respiratory control. Direct primary afferent input to the InM arises from upper cervical levels in a number of species [rat (Imamura et al. 1986; Neuhuber and Zenker 1989; Pfaller and Arvidsson 1988), Guinea pig (Prihoda et al. 1991), cat (Stechison and Saintcyr 1986) and monkey (Edney and Porter 1986)]. Outputs of neurones in the InM are relatively unknown—close examination of retrograde tracing from the hypoglossal nucleus suggests that this may be one target of InM neurones (Dauvergne et al. 2001; Popratiloff et al. 2001), but other connections within the CNS remain unidentified. Taken together, these studies are suggestive of a possible role for the InM in mediating autonomic/respiratory responses to sensory afferent signals arising from the upper neck.

Physiological studies are consistent with the notion that cervical sensory afferents influence autonomic function. In anaesthetised cat preparations, low threshold electrical stimulation of the second or third cervical nerve evokes activity in the hypoglossal and abdominal nerves, whilst either exciting or inhibiting sympathetic activity in the splanchnic nerve (Bolton et al. 1998). Following transection of the brainstem caudal to the vestibular nuclei, but rostral to the InM, the respiratory responses to upper cervical nerve stimulation were enhanced and a short latency response in sympathetic nerve activity was revealed. Interestingly, respiratory and sympathetic responses could be evoked from stimulation intensities that would only activate group I afferent fibres, highlighting a potential influence of proprioceptive afferents on cardiovascular and respiratory systems.

In this study we investigate whether upper cervical sensory afferents, particularly those arising from muscle spindle afferents, do indeed influence cardiorespiratory activity. To determine the pathways underlying responses to afferent stimulation we used neuronal tracing and immunohistochemical phenotyping to reveal the projections of neck muscle sensory afferents. The physiological properties of these projections were examined by stimulating the afferent pathways in brainstem slices. To investigate the potential central pathways processing the sensory signals we conducted anterograde neuronal tracing of central pathways from the InM. We then investigated the functionality of such pathways through direct chemical stimulation of the InM. Taken together our findings reveal cardiorespiratory responses to neck muscle afferent stimulation and central neuronal pathways that may be involved in mediating these responses.

## Methods

All experiments were performed under Home Office license, in accordance with the UK Animals (Scientific Procedures) Act, 1986 and according to local ethics.

### Neuronal tracing of afferent projections from C2 nerve

Adult male Wistar rats (250 g, *n* = 5) were anaesthetised with 5 % isoflurane in O_2_. A dorsal midline incision was then made from the base of the skull to the scapula. The trapezius muscle was then opened along the midline and the underlying muscle layers blunt dissected to gain access to the vertebral column. The nerve entering the C2 vertebra was then identified and 5 μl of cholera toxin b subunit (CTb, 1.5 % in 0.1 M PB, B. Turnbull University of Leeds) injected into the nerve using a glass micropipette. Following 3–5 days recovery, animals were anaesthetised with sodium pentobarbitone (60 mg/kg IP) and perfused transcardially with 500 ml 4 % paraformaldehyde (PFA). The brainstem and upper spinal cord were dissected and postfixed overnight in the same fixative. Tissue was then sectioned coronally at 50 μm and washed three times in PBS before incubation in goat anti CTb (1:10,000 in PBS containing 0.1 % triton X-100, List Biological) overnight at 4 °C. Sections were then washed three times in PBS before the tracer being visualised with a Cy3 conjugated secondary antibody raised against the goat IgG (1:500 in PBS, Jackson ImmunoResearch). To examine the phenotype of the traced afferents, sections were triple labelled for CTb, parvalbumin (PV) (mouse anti PV, 1:1,000 in PBS, Sigma) and vesicular glutamate transporter 1 (VGLUT1) (Guinea pig anti VGLUT1, 1:10,000 in PBS, Millipore). Following an overnight incubation in the second primary antibody at 4 °C sections were washed three times in PBS before incubation in Alexa Fluor^488^ conjugated secondary antibodies (donkey anti-mouse; 1:1,000 in PBS, Invitrogen) for 1 h at room temperature. Sections were then again washed in PBS before incubation in a biotinylated secondary antibody against the Guinea pig IgG (1:250 in PBS, Jackson ImmunoResearch) for 3 h at room temperature. Sections were then washed in PBS before a final incubation in streptavidin Pacific Blue (1:1,000 in PBS, Invitrogen). Co-localisation was then assessed using oil immersion ×100 objectives on a Nikon E600 microscope equipped with epifluorescence and appropriate filter sets.

### Neuronal tracing from the sternomastoid muscle (StM)

Adult male mice (*n* = 4) were deeply anaesthetised with 5 % isoflurane in O_2_. A ventral midline incision was made slightly rostral to the sternum and skin reflected to gain access to the StM. At approximately the midpoint of the muscle 5 μl of 1.5 % CTb was injected through a fine glass micropipette attached to a 10 μl Hamilton syringe. Following 3–5 days recovery animals were transcardially perfused with 4 % PFA, the brainstems and spinal cords postfixed and then sectioned at 50 μm. CTb was then detected using immunohistochemistry as above.

### Assessing InM populations for potential afferent inputs

Since all CTb-labelled structures within the InM displayed strong immunoreactivity for VGLUT1 and PV (and all observed VGLUT1 containing terminals within the InM also contained PV), VGLUT1 immunoreactivity was used as a marker of neck muscle afferent terminals. The degree of contact between VGLUT1 immunoreactive terminals and GABAergic neurones was investigated in tissue from transgenic mice expressing GFP under the control of the GAD65 (Lopez-Bendito et al. 2004) and GAD67 (Tamamaki et al. 2003) promoters. Adult wild-type and transgenic mice of both genotypes were anaesthetised with sodium pentobarbitone (60 mg/kg IP) and transcardially perfused with 200 ml 4 % PFA. The brainstems were then removed and postfixed overnight in the same perfusate. Coronal brainstem sections were then cut at 50 μm. Adult male rat tissue (*n* = 4) was also prepared by transcardial perfusion and sectioned as described above.

Brainstem sections from GAD65 and GAD67-GFP mice were incubated in Guinea pig anti-VGLUT1 (1:10,000 in PBS containing 0.1 % triton X-100, Millipore, UK) overnight at 4 °C. Sections then received three washes in PBS before incubation in biotinylated donkey anti-Guinea pig secondaries (1:250 in PBS, Jackson Immunoresearch, USA) for 4 h at room temperature. Sections were then washed three times in PBS prior to 1 h incubation in Streptavidin Alexa^555^ at room temperature. GFP fluorescence was enhanced through immunohistochemical detection of GFP using rabbit anti GFP (1:1,000 in PBS Invitrogen, UK) overnight at 4 °C. Sections were then washed three times in PBS prior to 2 h incubation in an Alexa Fluor^488^ conjugated donkey anti rabbit secondary antibody (1:1,000 in PBS, Invitrogen, UK). Sections were then washed three times in PBS before mounting in Vectashield (Vector Laboratories, USA) on glass microscope slides.

Wild-type mouse and rat brainstem sections were incubated in anti-VGLUT1 antibodies as described above in combination with antibodies against nNOS (Sheep anti nNOS 1:10,000, Dr P. Emson University of Cambridge), calretinin (goat anti CR; 1:5,000, SWANT, Switzerland) and PV (mouse anti PV, 1:1,000, Sigma, UK), diluted in PBS containing 0.1 % Triton X-100, as these neurochemicals have previously been demonstrated to be found within the InM (Edwards et al. 2007, 2009).

### Confocal microscopy

Sections were viewed on a Zeiss LSM510 confocal microscope using argon and HeNe lasers. Sections were viewed so that only cells, and not terminals, were visible to allow selection of cells wholly contained within the section for analysis without any bias towards the contact density. Once a cell had been selected it was optically sectioned at 0.3–0.5 μm and the resultant Z-stack exported from LSM image browser as a TIFF series.

The TIFF series was then imported into Reconstruct™, where a 3D reconstruction of the cell was created to estimate the cell’s surface area. The number of VGLUT1–IR terminals in close apposition, i.e., no black pixels can be observed between the stained terminals and somata, to the neurone was then counted.

### Electron microscopy

Adult male Wistar rats were anaesthetised using sodium pentobarbitone (60 mg/kg IP) and perfused transcardially with fixative containing 4 % PFA and 0.1–0.2 % glutaraldehyde (*n* = 3). Brainstems were removed and postfixed overnight in the same perfusate. Coronal brainstem sections were cut at 50 μm and collected into PBS. Sections were incubated in 50 % ethanol for 30 min to aid antibody penetration, before freeze fracturing in liquid nitrogen. Freeze-fractured sections were incubated in Guinea pig anti-VGLUT1 (1:10,000 in PBS, Millipore, UK) overnight at 4 °C and then in biotinylated donkey anti-Guinea pig secondaries (1:250 in PBS, Jackson ImmunoResearch, USA) for 4 h at room temperature, with three washes in PBS between each incubation. Sections were incubated in ExtrAvidin peroxidise (1:1,500 in PBS, Sigma, UK) overnight at 4 °C and visualised using diaminobenzidine as the chromogen. Stained sections were postfixed in 0.5 % osmium tetroxide (in 0.1 M PB) and dehydrated before embedding in Durcupan ACM resin (Fluka, Switzerland). Areas with suitable staining were prepared for electron microscopy and viewed on a Phillips CM10 transmission electron microscope. Negatives were digitised using an Epson 3200 Photo Perfection scanner.

### Antibody specificity

The goat anti CTb antibody (List Biological) has been widely used (Castillo-Ruiz et al. 2013; Messanvi et al. 2013; Milligan et al. 2006; Oka et al. 2013) to detect CTb following injection into central or peripheral targets. In tissue sections taken from animals which had not received injections of CTb, no immunoreactivity was observed.

The Guinea pig anti VGLUT1 antibody (Millipore) has been widely used for the specific detection of this isoform of the vesicular glutamate transporter (Aizawa et al. 2012; Atkinson et al. 2003; Brooke et al. 2006, 2010; Hughes et al. 2012; Milnerwood et al. 2012; She et al. 2012). It detects the expected protein size of 60 kDa in Western blots, and in immunohistochemistry shows complete overlap in rat brain with another well-characterised antibody against VGLUT1 (Melone et al. 2005).

The goat anti choline acetyl transferase (ChAT) antibody (Millipore) has been widely used and well characterised (Brooke et al. 2006; Jager et al. 2013; Milligan et al. 2006; Miyazaki et al. 2012; Muller et al. 2012; Yang et al. 2012). This antibody has been shown to detect a protein of 70 kDa when wild-type ChAT was expressed in COS cells (Ohno et al. 2001). In our hands, immunoreactivity has only observed within areas known to contain cholinergic neurones.

The mouse anti PV antibody (Sigma) binds to a single band with a molecular weight of 12 kDa in Western blots (Gabriel et al. 2004). This antibody has been widely used in the identification of interneurons in the hippocampus (Menegola et al. 2008), cerebellum (Ponti et al. 2008), cerebral cortex (Stevens et al. 2010) and brainstem (Edwards et al. 2007).

According to the manufacturers data sheet the goat anti CR antibody (SWANT) does not bind to tissue taken from CR knockout mice. In our hands this antibody gave strong binding in areas such as the striatum, where it is observed within a subpopulation of interneurones (Mura et al. 2000) and patterns of immunoreactivity were distinct to the other calcium binding proteins.

The sheep anti nNOS antibody (Dr P. Emson) detected a single protein with a mass of 155 kDa and preadsorption of the antibody with recombinant nNOS fully prevented immunolabelling in brain areas with high levels of nNOS expression (Herbison et al. 1996).

The rabbit anti GFP antibody (Invitrogen) displayed immunoreactivity in tissue taken from animals expressing GFP and never displayed immunoreactivity in wild-type animals. Further, detecting the GFP antibody using Alexa Fluor^555^ showed complete co-localisation with GFP.

All secondary antibodies were assessed for specificity in primary antibody omission controls, fluorescence was only observed when used in conjunction with the appropriate species of primary antibody.

### Image processing

Digital images were imported into Corel Draw X4 and brightness, contrast and intensity adjusted as appropriate.

### In vitro electrophysiology

Neonatal rats aged 13 days were terminally anaesthetised with urethane (2 mg/kg IP). The brain was dissected out and placed in ice cold aerated (95 % O_2_, 5 % CO_2_) sucrose artificial cerebrospinal fluid containing (aCSF, in mM) sucrose (217), NaCHO_3_ (26), KCl (3), MgSO_4_ (2), NaH_2_PO_4_ (2.5), CaCl_2_ (2) and glucose (10). 300 μm slices of the medulla oblongata were obtained using a Vibroslice and transferred to a holding chamber. The holding chamber contained aerated (95 % O_2_, 5 % CO_2_) aCSF (composition in mM): NaCl (124); NaHCO_3_ (26); KCl (3); MgSO_4_ (2); NaH_2_PO_4_ (2.5); CaCl_2_ (2); glucose (10).

Visualised patch clamp recordings were carried out at room temperature using an upright microscope (Olympus BX50WI; Optivision, Yorkshire, UK). Patch electrodes were filled with intracellular solution consisting of (in mM) K-gluconate (130), EGTA (11), MgCl_2_ (2), CaCl_2_ (1), HEPES (10), NaGTP (0.3), Na_2_ATP (2) and neurobiotin (0.05 %). Recordings were made in current clamp mode using an Axopatch ID (Axon Instruments, Foster City, CA) from neurones within the InM using standard procedures.

To investigate the presence of synaptic connections a bipolar stimulating electrode connected to an isolated stimulator (model DS2A; Digitimer, Hertfordshire, UK) was placed in the fibre tract lateral to the external cuneate nucleus and stimulated at twice the threshold for activation (duration 2 ms) to evoke post-synaptic potentials within InM neurones. To determine the nature of these synaptic potentials 2,3-dihydroxy-6-nitro-7-sulfamoyl-benzo[f]quinoxaline-2,3-dione (NBQX, 10 μM; Sigma, UK) was applied in the superfusate. All concentrations stated are the final drug concentration in the recording chamber.

Offline data analysis was carried out using Clampfit 9 software (Axon Instruments, Foster City, CA).

### Stereotaxic tracing of efferent projections from the InM

Under isoflurane anaesthesia (5 % in O_2_) stereotaxic injections were made into the InM of adult male Wistar rats (250 g, *n* = 5). Briefly the dorsal neck skin was incised along the midline and the underlying musculature blunt dissected to reveal the occipital bone and first cervical vertebrae, the occipital membrane was then incised. A 31G dental needle was connected to a 1 μl Hamilton syringe using PPE tubing and positioned over the InM [using co-ordinates taken from the rat brain atlas of Paxinos and Watson (1998); 1.0 mm rostral to Obex, 0.65 mm lateral to midline] using a Patchstar micromanipulator (Scientifica, Uckfield, UK) and slowly lowered to 1.3 mm. The dental needle was held in position for 30 min prior to injection of 40 nl of a 10 % solution of biotin dextran amine (BDA; 10,000 MW; Invitrogen, UK) dissolved in sterile H_2_O. The needle was then held in position for a further 30 min to allow the diffusion of pressure before retracting the injection needle to minimise back spill along the injection tract. Following recovery for 5–7 days animals were re-anaesthetised using sodium pentobarbitone (60 mg/kg IP) and transcardially perfused with 500 ml of 4 % PFA as above. Serial 50 μm sections of brainstem, and spinal cord were collected into PBS using a vibrating microtome as described above.

Within specific target, nuclei efferent tracing was visualised using Streptavidin Alexa^555^ (1:1,000 in PBS containing 0.1 % triton X-100) and combined with immunohistochemistry for either choline acetyl transferase (ChAT, goat anti ChAT, 1:500 in PBS, Millipore) or tyrosine hydroxylase (TH, sheep anti TH, 1:1,000, Abcam, UK), which were visualised with the appropriate Alexa Fluor 488 conjugated secondary antibody (1:1,000 in PBS, Invitrogen).

### Working heart brainstem preparation

21-day-old rats were prepared for the working heart brainstem preparation (WHBP) as previously described (Potts et al. 2000). Briefly, animals were anaesthetised with halothane, bisected subdiaphragmatically and decerebrated in ice cold Ringers solution. The preparation was then skinned, the phrenic nerve cleaned and isolated from the diaphragm and the hypoglossal nerve dissected free from the tongue. The dorsal neck region was dissected to reveal the C2 nerve as it leaves the vertebral column. The preparation was then transferred to the recording chamber, where the descending aorta was cannulated and perfusion flow rate set to ensure skull perfusion. Perfusion flow rate was altered to obtain a eupnic-like pattern of activity in the phrenic nerve and recordings were simultaneously taken from the hypoglossal nerve. In some experiments activity in the sympathetic chain was also recorded. In some experiments the StM was then vibrated at 50 Hz using a permanent magnet shaker (V203, Ling Dynamic Systems, UK), the sternomastoid was specifically chosen for these experiments as its unique orientation allows the specific vibration of this muscle without influencing any surrounding muscle groups. For nerve stimulation, the C2 nerve was transected close to the vertebral column and sucked into a stimulating electrode.

The stimulation intensity at the C2 nerve was set at the level required to cause a temporary cessation of phrenic nerve activity (PNA) in response to a single pulse. All stimuli were then delivered at this threshold. The C2 nerve was then stimulated at 5 or 10 Hz for 10 s. Phrenic nerve activity, hypoglossal nerve activity (HNA), perfusion pressure (PP) and heart rate (calculated from ECG recorded with PNA or from pulsatility of PP) were all recorded. Raw neurograms were subjected to rectification and integration, the integrated traces are presented alongside the raw neurograms for clarity.

For microinjection into the InM, double-barrelled micropipettes were stereotaxically lowered into the InM and 40 nl of 100 mM glutamate or 20 mM NBQX pressure ejected. Injection sites were confirmed histologically.

For analysis, bouts of PNA were only considered to be inspiratory bursts when they displayed an incrementing ramp-like discharge pattern. Levels of SNA were calculated from the area under the curve of the integrated SNA trace. Calculations were made from a timebase long enough to include a full respiratory cycle, which was compared to the average activity of an equivalent timebase before and after the stimulation period.

### Data presentation

All numerical data are presented as mean ± standard error of the mean, unless stated otherwise. *N* values refer to animals whilst *n* refers to the number of cells. Statistical significance was tested using ANOVA with a post hoc Tukey’s test using Minitab 15, or paired *t* test using MS Excel 2007.

## Results

### Cardiovascular and respiratory responses to sternomastoid muscle vibration and C2 nerve stimulation

To determine whether the stimulation of muscle sensory afferents could influence cardiovascular and respiratory activities, we vibrated the StM or electrically stimulated the C2 nerve in the WHBP.

**Fig. 1:**
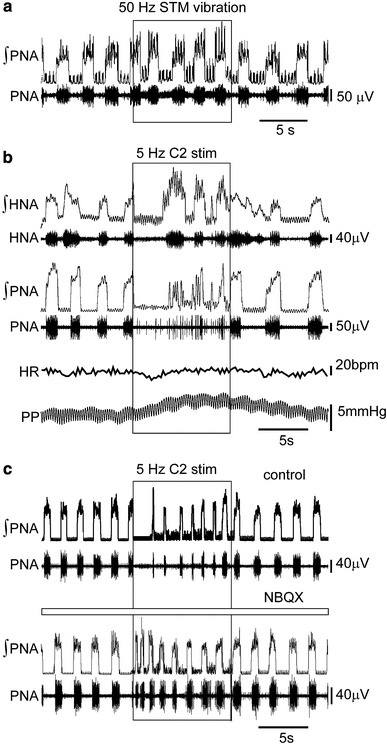
Muscle spindle activation or electrical stimulation of the second cervical nerve evokes tonic hypoglossal nerve discharge, reduces inspiratory drive and increases vascular resistance. **a** Vibration of the sternomastoid muscle (STM) at 50 Hz caused a small decrease in the duration of inspiratory bursts during the stimulus (*boxed area*), which returned to normal upon cessation of the vibration. **b** Electrical stimulation of the C2 nerve at 5 Hz evokes tonic discharges in HNA, during these bouts of tonic discharge PNA is suppressed—both traces are accompanied by rectified and integrated traces (∫HNA and ∫PNA). Over the stimulation period, perfusion pressure (PP) rises with little effect upon heart rate (HR). **c** The suppression of PNA through 5 Hz stimulation of the C2 nerve is markedly reduced following injection of NBQX into the InM

Vibrating the sternomastoid (1 mm displacement, 50 Hz) evoked a small decrease in the duration of phrenic bursts from 854 ± 56 to 791 ± 42 ms (*N* = 4, *p* = 0.03, Fig. 1a), with no significant changes in the expiratory period or total respiratory period. Vibration of the StM following detachment from the sternum or vibrating in the vicinity of the StM following complete removal of the muscle had no effect upon any respiratory or cardiovascular parameters. Stimulation of the C2 nerve at 5 Hz suppressed PNA to a greater extent. During stimulation there was a long quiescent period with brief escapes from the inhibition, evident as short bursts of activity without the typical incrementing pattern associated with inspiration. When a true ramping pattern of activity was observed in the phrenic nerve during the stimulation, the inspiratory period of the respiratory cycle (Ti) reduced significantly from 766 ± 140 to 490 ± 144 ms (*N* = 5, *p* = 0.003). In contrast, C2 stimulation elicited tonic HNA which had no correlation with the respiratory cycle. Overlying the tonic activity inspiratory related bursts could still be observed, which correlated with PNA. During the stimulation there was a small, but highly significant (*p* = 0.003) increase in perfusion pressure of 1.9 ± 0.5 mmHg (*N* = 5), without a significant change in heart rate (Fig. 1b).

In order to determine if the strong inhibition of PNA was mediated through C2 nerve inputs to the InM, the C2 nerve was stimulated in the presence of NBQX stereotaxically injected into the InM. There was a striking reduction in latency to the first sustained PNA—prior to injection with NBQX the time from the onset of 10 Hz stimulus of the C2 nerve to the first sustained PNA was 3.38 ± 1.13 s, following the injection of NBQX into the InM this latency was significantly reduced to 1.50 ± 0.65 s (*N* = 4, *p* = 0.03, Fig. 1c).

### C2 muscle afferents terminate within the intermedius nucleus of the medulla (InM)

**Fig. 2:**
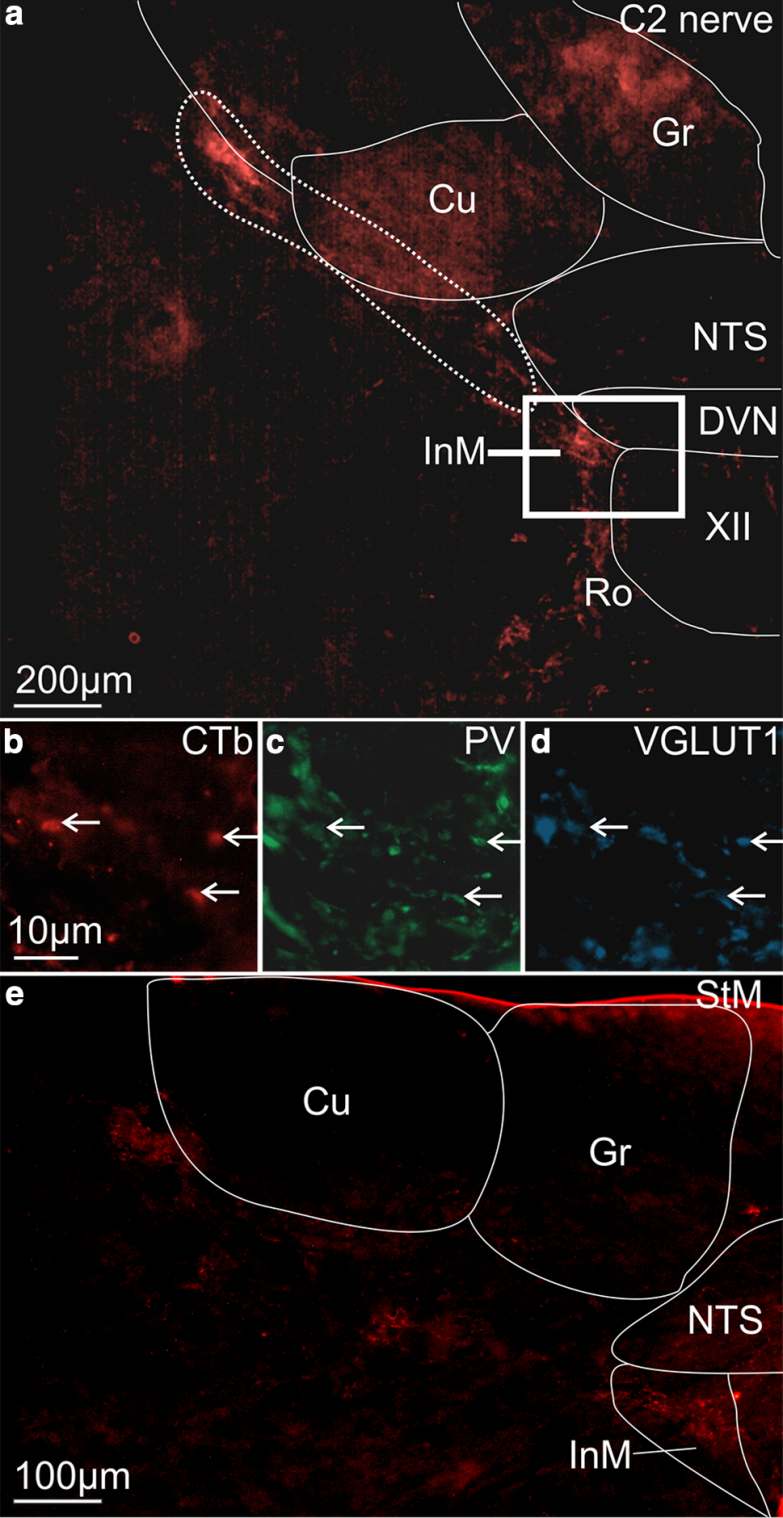
Afferents traced from C2 to the InM have a proprioceptive origin. **a** CTb immunoreactivity in the brainstem after injection into the C2 nerve. The area marked with a *dashed line* highlights the strong band of fibres coursing towards the InM. **b** High power view of CTb immunoreactivity in the InM, *scale bar* also applies to **c** and **d**. **c** Parvalbumin immunoreactivity in the same 50 μm section. **d** VGLUT1 immunoreactivity in the same 50 μm section. Examples of triple labelled terminals are indicated with *arrows*. **e** Afferent fibres traced from the sternomastoid muscle follow the same brainstem trajectory as those traced from the C2 nerve

In order to further clarify the sensory modality of upper cervical primary afferent input to the InM, the central trajectory of primary afferents from the second cervical nerve was traced using CTb. Within the cervical spinal cord CTb immunoreactivity could be seen within the cuneate fascicle of the dorsal columns. Presumed afferent terminals could be seen within the deep dorsal horn laminae, with a concentration around PV immunoreactive neurones in the central cervical nucleus. Little or no afferent staining was observed within the superficial dorsal horn. Within the brainstem, CTb immunoreactivity was detected in a distinct fibre bundle travelling ventro-medially from the dorso-lateral border of the external cuneate nucleus towards the perihypoglossal nuclei (Fig. 2a). A strong pattern of terminal-like labelling was observed within the InM (Fig. 2a). Varying the survival time from 3 to 5 days did not alter the pattern of staining.

Traced afferents within the InM were identified as having a likely muscle origin by co-staining for CTb and PV and/or VGLUT1, both of which are reliable markers for muscle afferents (Alvarez et al. 2004; Arber et al. 2000). The vast majority of the CTb-labelled structures within the InM displayed immunoreactivity for VGLUT1 (91.6 ± 5.1 %, 110/126, *n* = 4) or PV (82.9 ± 5.0 %, 95/124, *n* = 4), see Fig. 2b–d. Clearly, not all VGLUT1 immunopositive structures within the InM were CTb labelled since only one dorsal root at one level was injected and dorsal roots at C1–C4 all project to the InM (Neuhuber and Zenker 1989; Stechison and Saintcyr 1986).

The muscle origin of the projections to the InM was then further confirmed by tracing the afferent projections of the StM, which projects to the CNS via the second cervical sensory root. When tracer was applied to the StM primary afferent labelling was observed within the brainstem with an identical trajectory to that observed when tracing from the whole nerve, although with a noticeable reduction in the density of stained fibres. As with tracing from the whole nerve strong terminal labelling was observed within the InM (Fig. 2e).

### All neuronal phenotypes within the InM are contacted by VGLUT1 containing, presumed primary afferent, terminals

Anterogradely traced afferents within the InM contained both VGLUT1 and PV, previously shown to be enriched in muscle afferents (Alvarez et al. 2004; Arber et al. 2000). Furthermore the VGLUT1 terminals which did not contain CTb also contained PV, consistent with them being sensory afferents also with a muscle origin. As primary afferents from the first 4 cervical DRG all project to the InM (Edney and Porter 1986; Imamura et al. 1986; Neuhuber and Zenker 1989; Pfaller and Arvidsson 1988; Prihoda et al. 1991; Stechison and Saintcyr 1986) it is highly likely that these untraced VGLUT1 terminals arise from one of the other upper cervical sensory ganglia. Therefore, as reported within the dorsal horn (Todd et al. 2003), within the InM VGLUT1 appears to be a robust marker of primary afferents with a muscle origin. We therefore used the presence of VGLUT1 in terminals to assess whether different neuronal populations within the InM have the potential to receive primary afferent inputs.

We have previously shown the InM to be a neurochemically rich nucleus (Edwards et al. 2007, 2009) and characterised the expression of the calcium binding proteins PV and calretinin (CR), and the neurotransmitter synthesising enzymes neuronal nitric oxide synthase (nNOS) and glutamic acid decarboxylase (GAD) 65 and 67 as well as VGLUT2 within this nucleus. These populations are not mutually exclusive; indeed the CR immunoreactive population comprises approximately 95 % of the GFP expressing cells in VGLUT2-GFP mice. We therefore sought to assess the degree of connectivity between VGLUT1 containing terminals and the different neuronal populations within the InM.

**Fig. 3:**
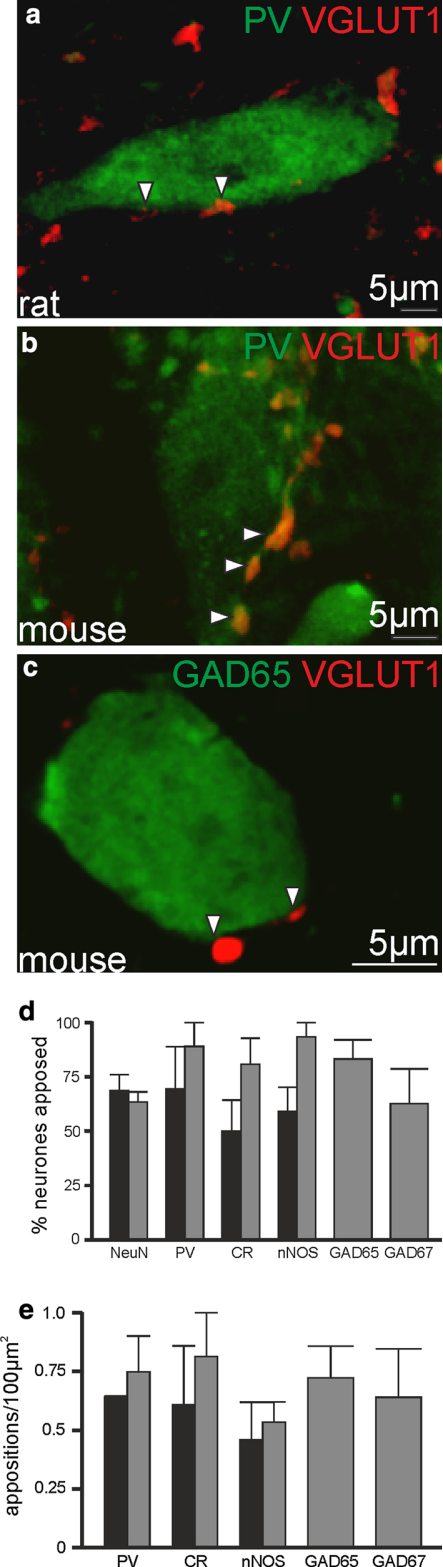
InM neurones are targeted with VGLUT1 terminals irrespective of their phenotype. **a** A parvalbumin immunoreactive neurone in the InM of the rat in close apposition to VGLUT1 terminals. **b** A parvalbumin immunoreactive neurone in the InM of the mouse in close apposition to VGLUT1 terminals. **c** An example of a GFP expressing neurone in the InM from GAD65-GFP mice in close apposition to VGLUT1 terminals. **d** Group data showing little variance in the proportion of neurones seen in close appositions to VGLUT1 terminals. **e** Group data showing little variance in the density of appositions onto InM neurones. *Black bars* in **d** and **e** are data from rat, *grey bars* are from mouse

Using NeuN as a pan neuronal marker, VGLUT1 immunoreactive structures were found in close apposition to 68.6 ± 7.4 % of NeuN immunoreactive neurones within the rat InM (*n* = 44/68, *N* = 3). Further dividing this to different neuronal phenotypes, 69.4 ± 19.4 % (*n* = 15/22, *N* = 3), 50 ± 14.4 (*n* = 9/18, *N* = 3), and 59.1 ± 11.2 (*n* = 12/21, *N* = 3) % of neurones immunoreactive for PV, CR and nNOS, respectively, had VGLUT1 terminals in close apposition (an example of VGLUT1 immunoreactive terminals in close apposition to a rat PV neurone is shown in Fig. 3a, group data are summarised in Fig. 3d). No significant differences were observed between the populations. Similarly, the density of contacts surrounding the somata of stained neurones was not significantly different between the populations; with 0.64 ± 0.007, 0.61 ± 0.25, and 0.46 ± 0.16 contacts per 100 μm^2^ of cell membrane for PV, CR and nNOS, respectively, (summarised in Fig. 3e).

To determine the connectivity between VGLUT1 terminals and GABAergic neurones, we used the previously described GAD65- and GAD67-GFP mouse lines (Lopez-Bendito et al. 2004; Tamamaki et al. 2003). To clarify any species differences, the experiments detailed above were repeated in the mouse. 63.4 ± 4.7 % (*n* = 8/13, *N* = 3) of NeuN immunoreactive neurones in the InM received close appositions from VGLUT1 containing terminals, which was not significantly different to that seen in the rat. 88.9 ± 11.1 (*n* = 8/9, *N* = 3) of PV, 80.2 ± 12.4 (*n* = 15/19, *N* = 3) of CR and 93.3 ± 6.7 (*n* = 21/22, *N* = 3)  % of nNOS + ve populations receiving close appositions, respectively (an example of a mouse PV neurone receiving close appositions from VGLUT1 immunoreactive terminals is shown in Fig. 3b, and group data is summarised in Fig. 3d). 83.07 ± 8.66 (*n* = 23/28, *N* = 3) and 62.5 ± 15.8 (*n* = 9/14, *N* = 3) % of GFP expressing neurones in the GAD65-GFP and GAD67-GFP lines received close appositions from VGLUT1 containing terminals (an example of a GAD65-GFP neurone receiving close appositions from VGLUT1 immunoreactive terminals is shown in Fig. 3c). Again no significant differences could be detected in the density of contacts surrounding the membranes of each of these populations (PV 0.75 ± 0.15, CR 0.81 ± 0.19, nNOS 0.53 ± 0.13, GAD65-GFP 0.72 ± 0.13 and GAD67-GFP 0.64 ± 0.2 contacts per 100 μm^2^, *N* = 3 for each, summarised in Fig. 3e).

**Fig. 4:**
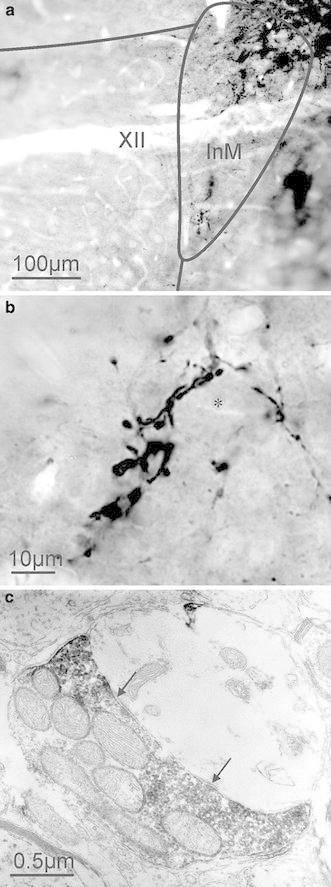
Ultrastructural analysis of VGLUT1 immunoreactive structures within the InM. **a** VGLUT1 immunoreactivity is strong within the InM. **b** Higher magnification view of VGLUT1 immunoreactive structures in the InM, some VGLUT1 containing structures appear to be closely associated with unstained InM neurones (marked with *). **c** VGLUT1 immunoreactivity was only observed within axon terminals, which can be seen to form synapses (marked by *arrows*) with unlabelled dendrites

We verified that the terminals marked with VGLUT1 do indeed form synapses in the InM using electron microscopic analysis of VGLUT1 immunoreactive structures. VGLUT1-labelled terminals typically contained small clear vesicles and formed synaptic contacts with neurones within the InM (*n* = 28 terminals analysed, *N* = 3; Fig. 4).

**Fig. 5:**
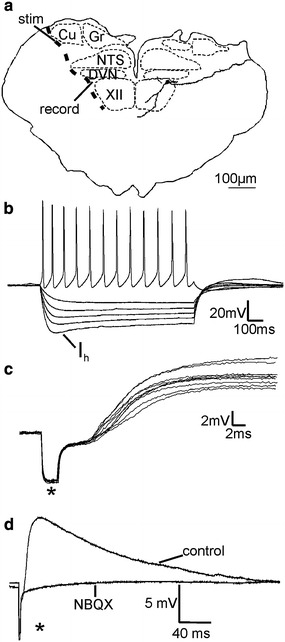
Stimulation of the fibre pathway lateral to the external cuneate nucleus elicits in monosynaptic EPSPs in InM neurones. **a** Cartoon showing the course of primary afferent fibres relative to the stimulation and recording sites used here. Superimposed upon the right hand side of the cartoon is a camera lucida drawing of an InM neurone. **b** Most InM neurones display a small Ih and all fire a train of action potentials in response to depolarising pulses. **c** 10 consecutive EPSPs in a single InM neurone showing minimal synaptic jitter. **d** EPSPs evoked through stimulation of the afferent pathway coursing lateral to the cuneate nucleus evokes are blocked by the non-NMDA excitatory amino acid glutamate receptor antagonist NBQX. Traces shown are an average of ten consecutive sweeps. Stimulus artefacts are marked with *asterisks*

Functional verification of afferent synaptic inputs onto InM neurones was then explored using electrophysiology. In slices of brainstem, InM neurones were recorded in whole-cell patch clamp configuration whilst an electrical stimulus was applied to the lateral border of the cuneate nucleus in a position corresponding to where the fibres traced from the C2 dorsal root and STM were observed through neuronal tracing (Fig. 2a, e). In unpublished experiments looking at brainstem areas providing inputs to the InM following injection of retrograde tracers into the nucleus we have never observed labelled neurones in the region of the dorsal column nuclei, minimising the possibility that the stimulation is activating a direct pathway from the cuneate nucleus to the InM. Neurones in the InM had a resting membrane potential of −42 ± 3.2 mV, an input resistance of 665 ± 53 MΩ (measured at −60 mV) and fired tonically upon depolarisation (*n* = 21). Action potentials in InM neurones in response to a +40 pA current pulse had an amplitude of 54.7 ± 1.6 mV, a half-width of 4.2 ± 0.3 ms, and displayed an after hyperpolarisation of 13.5 ± 1.0 mV. Hyperpolarising pulses of −100 pA evoked a voltage sag typical of the current I_h_ with an amplitude of 9.1 ± 1.3 mV in most neurones (16/21). Examples of firing properties of InM neurones are also shown in Fig. 5b. Recorded neurones were filled with neurobiotin and rhodamine and their location in the InM confirmed post hoc. Filled cells were spindle shaped with soma size 24.3 ± 2.9 by 14.7 ± 2.2 μm (Fig. 5a, *n* = 11). Electrical stimulation elicited excitatory post-synaptic potentials (EPSPs) in all InM neurones tested (*n* = 13) with minimal synaptic jitter (105 ± 27 μs, Fig. 5c) indicative of a monosynaptic pathway from the stimulation site to the InM. High-frequency stimulation of the fibre tract evoked a train of EPSPs within InM neurones with high fidelity and no failures, a feature often observed in primary afferent terminals. EPSPs had a latency of 6.7 ± 0.43 ms, an amplitude of 6.1 ± 0.69 mV, a half-width of 69.2 ± 16.17 ms and a 10–90 % rise rate of 1.2 ± 0.3 mV/ms (*n* = 13). The non-NMDA excitatory amino acid receptor antagonist NBQX (10 μM) significantly reduced the mean amplitude from 5.8 ± 1.0 mV to 1.1 ± 0.3 mV (*n* = 8, *p* < 0.001), see Fig. 5d.

### Neurones in the InM send efferent projections to known CNS areas associated with cardiovascular and oromotor functions

**Fig. 6:**
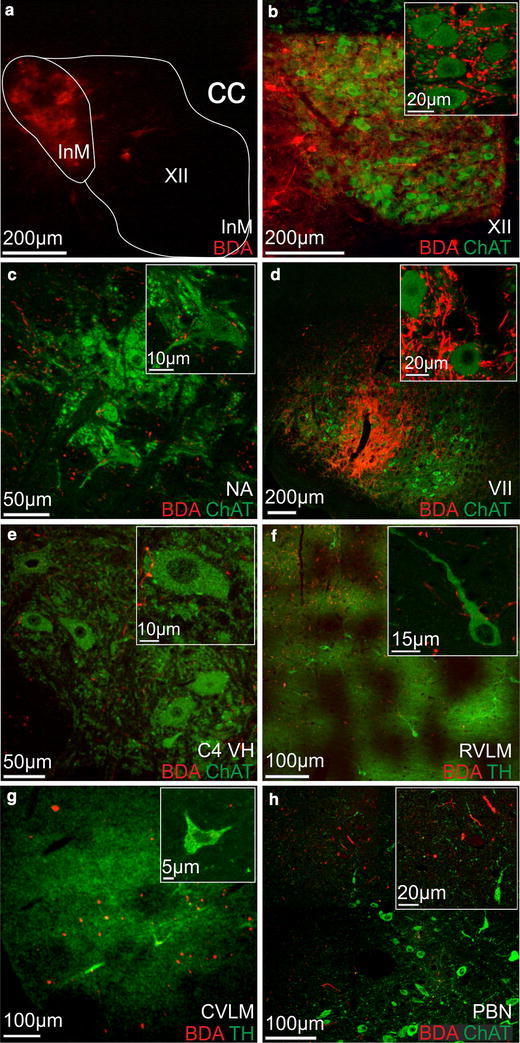
Anterograde projections from the InM. **a** Biotin dextran amine (BDA) deposited in the InM. **b** BDA labelling (*red*) throughout the XII. Labelled fibres closely appose ChAT positive XII motoneurones (*green*, see *inset*). **c** BDA-labelled efferents (*red*) apposing motoneurones within the semi compact formation of nucleus ambiguus (NA). **d** BDA-labelled efferents (*red*) apposing motoneurones (*green*) in the central third of the facial nucleus (VII). **e** BDA-labelled efferents (*red*) apposing presumed phrenic motoneurones in the cervical spinal cord (C4 VH). **f** InM efferents (*red*) within the rostral ventrolateral medulla (RVLM) close to tyrosine hydroxylase positive (TH, *green*) C1 cells. *Inset* shows TH immunoreactive neurones apposed by InM efferents. **g** InM efferents (*red*) in the caudal ventrolateral medulla (CVLM) close to the A1 cell group, identified by TH (*green*). TH immunoreactive A1 neurones do not appear to be targeted by InM efferents. **h** InM projections (*red*) within the parabrachial nucleus (PBN), dorso-lateral to the cholinergic neurones (ChAT, *green*)

To determine whether InM neurones project to regions of the CNS with known cardiovascular or oromotor functions, injections of the anterograde tracer BDA were made into the InM (*n* = 5, Fig. 6) and the efferent projections from this nucleus were then mapped according to the brain maps of Paxinos and Watson (Paxinos and Watson 1998). The small dimensions of the InM mean that there is a degree of tracer spill into some of the neighbouring nuclei, but the pattern of efferent staining remained the same regardless of which other nuclei also took up small quantities of the BDA. Furthermore, no evidence of BDA transport along the XII or X roots was observed. Within all of the experimental animals efferent projections from the InM were bilateral with an ipsilateral predominance. The primary efferent target from the InM appears to be the neighbouring XII, in which fibres and puncta could be observed bilaterally throughout its entire rostro-caudal extent. Another major target of the InM is the facial nucleus, which again displayed a bilateral projection confined to the central third of the nucleus. InM efferents could also be observed in the rostral and caudal divisions of the ventrolateral medulla, coursing through the intermediate reticular formation with terminals observed within the nucleus ambiguus. Efferent projections were observed in the NTS, with a stronger concentration of efferents observed within the rostral NTS, predominantly within the medial subnucleus. In more rostral brainstem sections InM efferents could be seen within the caudal portion of the spinal vestibular nucleus.

To further clarify the targets of the InM efferent projections, BDA labelling was combined with immunohistochemistry for ChAT or TH to label either motoneurones or to identify the catecholaminergic cell groups in the ventrolateral medulla. Again, whilst some of the tracer deposit was clearly within the hypoglossal nucleus (XII), little or no BDA appeared to be taken up by XII motoneurones due to the absence of any BDA-labelled ChAT immunoreactive neurones in the XII (see Fig. 6b). InM efferents could be observed bilaterally throughout the rostro-caudal extent of the XII in close apposition to motoneurones displaying ChAT immunoreactivity (Fig. 6b). At these levels InM efferents could also be observed in close apposition to ChAT immunoreactive neurones within the semi compact portion of the nucleus ambiguus (NA), with little or no projection into the compact NA (Fig. 6c).

At more rostral levels of brainstem a second strong projection can be seen entering the facial nucleus (VII). Again these projections are bilateral, and appear to be concentrated to the central third of the nucleus where they closely appose ChAT immunoreactive motoneurones (Fig. 6d).

BDA-traced efferent projections from the InM were also seen in close apposition to ventral horn motoneurones in the cervical spinal cord at levels C4–6, predominantly around presumed phrenic motoneurones located more medially in the ventral horn (Fig. 6e).

Clear projections from the InM into the caudal and rostral regions of the ventrolateral medulla can also be observed, although these projections are not as strong as those observed in the motor areas. Within these regions the traced efferents are not closely associated with TH immunoreactive neurones of the C1 and A1 cell groups (Fig. 6f, g).

Within the pons, traced efferents could be observed near the dorsal surface within the parabrachial nucleus, although typically more dorsal and lateral to the cholinergic neurones therein (Fig. 6h).

### Direct stimulation of the InM increases HNA asynchronous to PNA and evokes an increase in perfusion pressure, likely sympathetically mediated

**Fig. 7:**
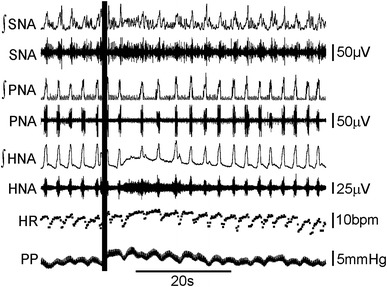
Microinjection of glutamate into the InM causes respiratory depression, sympathoexcitation and HNA activation. Microinjection of glutamate (40 nl of 100 mM) into the InM (injection sites confirmed histologically using pontamine sky blue) at the time marked by the *vertical line* evokes tonic activity in HNA and ∫HNA, with underlying inspiratory activity. Following glutamate injection the expiratory period in PNA and ∫PNA was exaggerated and SNA and ∫SNA became more tonic, with a cessation in respiratory related bursts. This is coupled to a small increase in PP with no obvious changes to HR, other than diminished respiratory sinus arrhythmia

As efferent projections from the InM were observed within cardiovascular, respiratory and oromotor regions of the brainstem, we performed direct chemical stimulation of the nucleus in the WHBP to investigate whether this would alter cardiorespiratory output. Microinjection of 20–40 nl of 100 mM glutamate into the InM (Fig. 7, *n* = 5) reduced PNA, with a small reduction in the inspiratory period (618 ± 33 to 546 ± 49 ms) and an increase in the expiratory period (1,903 ± 188 to 8,599 ± 4,706 ms), resulting in a significant reduction in the recorded breaths per minute from 25.2 ± 2.5 to 12.9 ± 2.7 (*p* < 0.05, *n* = 5). Injection of glutamate into the InM introduced a tonic discharge in HNA, through which inspiratory modulation could sometimes still be observed. Chemical stimulation of the InM also evoked a short small increase in perfusion pressure of 1.49 ± 0.9 mmHg, which was similar to that observed when stimulating the C2 nerve. This was accompanied by a tonic discharge of sympathetic nerve activity (SNA), with an overall increase to 108 ± 12.8 % of baseline levels (*n* = 5). All other respiratory parameters had returned to their baseline levels once the tonic HNA had subsided.

## Discussion

This study shows that stimulation of neck muscle afferents can influence behaviour of the cardiovascular and respiratory systems. This is due to unique neuronal pathways which exhibit specialised functions, the first stage of this signalling arising from neck muscles. Neurophysiological and neuroanatomical experiments indicate that the sensory input exerts its influence via the InM, which appears to act as an integratory centre.

### Neck muscle afferent input to the intermedius nucleus of the medulla (InM)

Neuroanatomical studies looking at the central targets of cervical sensory afferents have revealed a direct sensory projection to the InM from upper cervical (Edney and Porter 1986; Imamura et al. 1986; Neuhuber and Zenker 1989; Pfaller and Arvidsson 1988; Prihoda et al. 1991; Stechison and Saintcyr 1986), but not lower cervical levels (Edney and Porter 1986; Neuhuber and Zenker 1989; Stechison and Saintcyr 1986). The input to the InM is thought to be of a muscle origin as when tracers are applied to the lesser occipital nerve, a pure skin nerve branch of C2, no terminal labelling is observed within the InM; however, if the tracer is injected into the StM which is also innervated from C2, the terminal labelling is still evident in the InM. Furthermore, the staining pattern from injection of the tracer into the whole nerve very closely resembles the staining pattern observed when the tracer is applied directly to one of the neck muscles (Neuhuber and Zenker 1989). This suggests that the majority of the traffic to the InM through the C2 nerve is likely to be of a muscle origin. Here we report that within the spinal cord sensory afferents traced from the second cervical nerve target the deeper dorsal horn laminae and the central cervical nucleus, with little or no innervation of the superficial dorsal horn. Indeed, consistent with previous tracing studies where the tracer has been applied directly to neck muscles (Edney and Porter 1986; Neuhuber and Zenker 1989; Pfister and Zenker 1984) we have shown many of the primary afferent inputs to the InM arise from the musculature of the neck. Interestingly, the muscles supplying the upper cervical segments are particularly rich in muscle spindles (Kulkarni et al. 2001; Richmond and Abrahams 1975), up to threefold higher than that observed in hindlimb muscles.

Our results suggest specialisation of neck muscle spindle pathways versus those of limb muscles since activation of the upper cervical afferents suppressed PNA with increase in perfusion pressure, whilst stimulation of the brachial plexus, which projects to the CNS via the lower cervical dorsal root ganglia and contains the afferent fibres from forelimb muscles, evokes activity in the phrenic nerve with a concurrent increase in perfusion pressure and heart rate (Potts et al. 2000). As the InM does not receive inputs from primary afferents caudal to C4 (Edney and Porter 1986; Neuhuber and Zenker 1989; Stechison and Saintcyr 1986), even though sensory afferents from all cervical dorsal root ganglia ramify in several other common CNS areas such as the spinal dorsal horn, the central cervical nucleus, cuneate nucleus and vestibular nuclei, it is highly possible that the specialised response to upper cervical stimulation occurs through pathways including the InM. Furthermore, the reduction in PNA coupled to an increase in perfusion pressure was also observed through direct stimulation of the InM, strengthening the role of InM in mediating specific actions of neck muscle afferents on oromotor, respiratory and sympathetic systems.

Taking advantage of the anatomical organisation of the brainstem we have shown that the InM directly responds to electrical stimulation of primary afferent pathways in brainstem slices. In these slice preparations every single InM neurone responded with monosynaptic EPSPs from stimulation of the lateral border of the external cuneate nucleus. In retrograde tracing studies, large deposits of tracer were injected into the InM; in these studies no retrogradely labelled neurones were ever observed in either of the dorsal column nuclei (Edwards unpublished observations), reducing the possibility that the stimulus was actually activating a hitherto unknown connection from the dorsal column nuclei to the InM.

The 100 % success rate for EPSPs in InM neurones following primary afferent pathway stimulation correlates well with almost 70 % of every neurochemically defined population of InM neurones receiving close appositions from VGLUT1 containing terminals. The discrepancy between these values may be due to limiting the assessment of appositions to the somatic region. However, the immunoreactivity pattern for the antigens used here did not allow fair comparisons of the dendritic profiles. There is also the possibility that the VGLUT1 containing terminals do not all have a neck muscle origin. However, VGLUT1 is preferentially found within centrally projecting peripheral neurones rather than central neurones and it co-localises with markers of sensory mechanoreceptors (Alvarez et al. 2004). Within the InM all VGLUT1 containing terminals also contained PV, which is often used as a marker of sensory afferents with a muscle origin (Alvarez et al. 2004; Arber et al. 2000). As many VGLUT1 terminals within the InM could be directly traced to a C2 origin, and all VGLUT1 containing terminals in this region displayed markers of muscle sensory afferents it is most likely that the VGLUT1 containing terminals within the InM all have a muscle origin. These muscles are likely to be neck muscles as only afferents arising from C1–4 have been demonstrated to project to the InM of the rat (Imamura et al. 1986; Neuhuber and Zenker 1989; Pfaller and Arvidsson 1988). Another reason for discrepancy may be the result of the different age of animals used for electrophysiology and anatomy experiments, resulting in an over representation of this pathway in the younger animals. Due to increased levels of myelination and the reduction in viability of brainstem slices taken from older animals these experiments have to be performed at an age when synaptic connections are still maturing. However, it is important to note that the connection between the sensory afferents in question and InM neurones do clearly persist into adulthood as demonstrated by the tracing experiments.

Previously we have used mice expressing GFP under the control of the VGLUT2 promoter to investigate the neurochemistry of glutamatergic neurones in the InM (Edwards et al. 2009). Due to the mosaic expression pattern too few GFP expressing neurones were evident within the InM to assess potential contacts between VGLUT1 terminals and glutamatergic neurones. However, the CR immunoreactive population of InM neurones almost entirely accounted for the glutamatergic neurones in the VGLUT2 GFP mice (Edwards et al. 2009). Therefore, as the CR immunoreactive neurones receive approximately the same number and density of VGLUT1 appositions as any of the other populations it is fairly safe to assume that glutamatergic neurones within the InM are also targeted by neck muscle afferent inputs. It is therefore also interesting to note that the GABAergic population of neurones within the InM (whether GAD65 or GAD67 containing) are also targeted at approximately the same level. The indiscriminate targeting of presumed neck muscle afferents within the InM raises the possibility of this nucleus acting as a sensory relay station although it will be interesting for future studies to determine if particular muscle groups and their antagonists share the same innervation patterns.

### Efferent tracing from the InM

This first report of the efferent projections from the InM took advantage of the relative lack of specificity of BDA to identify as many target structures as possible. This, of course, harboured the risk of tracing projections from neighbouring nuclei and as such we deliberately tried to centre the injection site over the most medial aspect of the nucleus constraining tracer spill to the XII (the projections from which could have easily been identified as being cholinergic projections travelling ventrolaterally through the brainstem to form the XII root). The InM projections throughout the XII were not the result of tracing from the XII itself as the 10,000 molecular weight BDA used here travels predominantly anterogradely and was never co-localised with ChAT immunoreactivity, indicating that it was not contained in potential motoneurone collaterals.

There is also the risk of back spill of tracer along the injection track into the NTS, and as such we disregarded any labelling observed within this nucleus at the level of the injection site. Importantly, in all of the tracing experiments performed there was no evidence of tracing along vagal efferents, and only weak projections to the rostral ventrolateral medulla and nucleus ambiguus which are heavily targeted by the NTS (Ross et al. 1985).

### Is the InM a gateway to the hypoglossal motor nucleus?

Efferent labelling in XII following InM injections was very dense, indicating that the InM is a major source of input to the hypoglossal nucleus. Further, activation of the InM directly or through stimulation of upper cervical sensory afferents elicits a tonic discharge in HNA. It is also possible that cervical sensory afferents directly impinge upon the dendrites of XII motoneurones which course close to the InM (Milligan et al. 2006), although this has never been reported and the afferents we traced did not follow the mediolateral orientation of the XII dendrites, but rather matched those of the InM neurone morphology (See Fig. 5a). In addition, several important regions that influence tongue function do not project directly to the hypoglossal nucleus but use unidentified intermediaries [e.g. motor cortical tongue area (Alipour et al. 2002), jaw muscle spindle afferents (Nomura and Mizuno 1985) and NTS (Beckman and Whitehead 1991)]. In some cases these interceding cells may be InM neurones since terminals labelled anterogradely from the projecting areas can be found in the vicinity of the InM [e.g. motorcortical tongue area (Alipour et al. 2002), NTS (Beckman and Whitehead 1991)]. Whilst it has yet to be shown that these projections terminate upon the same InM neurones that innervate XII, the InM appears to be in a prime position to integrate information from both the periphery and the CNS before influencing airway patency and tongue movements through the hypoglossal nucleus.

### Cardiorespiratory responses to neck afferent stimulation

Here we report that electrical stimulation of the second cervical nerve has a suppressive effect upon inspiratory activity in the phrenic nerve, whilst also evoking a tonic discharge pattern in the hypoglossal nerve. Whilst the stimulation parameters used here would likely activate all classes of fibre, we have also demonstrated that the predominant fibre type within this nerve corresponds to muscle afferents. One of the main targets of this input that appears to be unique to the upper cervical sensory afferents is the InM. Direct stimulation of the InM with glutamate mimics the response to whole peripheral nerve stimulation, highlighting that this nucleus plays an integral part in the responses measured to upper cervical peripheral nerve stimulation.

In response to stimulating the C2 nerve or the InM we observed a pressor response, which upon direct stimulation of the InM correlates with an increase in SNA, with no obvious heart rate response coupled to a suppression of inspiratory drive. Given that the stimulation parameters used in this study were quite broad we may have activated any or all of the different fibre types within the C2 nerve. The similarity of the responses observed when stimulating the C2 nerve and the InM directly strongly suggests that the response is mediated through pathways involving the InM, which specifically utilises muscle afferents. This response is different to that observed after nociceptive stimulation of the forelimb. Paw pinch and injection of inflammatory mediators into the forelimb all cause a decrease in respiratory cycle length coupled to tachycardia and an increase in perfusion pressure in the WHBP (Boscan and Paton 2002). The upper cervical response also differs to activation of mechanosensitive afferents in the forelimb through muscle contraction, which also presents with an increased respiratory frequency and a tachycardic pressor response (Potts et al. 2000). We would therefore argue that the unique response of respiratory depression with a sympathetic pressor response is a direct result of stimulating pathways which also incorporate the InM, through the activation of upper cervical muscle afferents.

Sympathetic and respiratory responses to stimulation of the second cervical nerve have also been shown in decerebrate cats. C2 stimulation in these animals, at intensities which should only activate proprioceptive afferents, also elicited an increase in splanchnic sympathetic nerve activity (Bolton and Ray 2000), whilst stimulating the hypoglossal and suppressing abdominal nerve activity. These responses persisted, or were in some cases amplified, after transection of the brainstem caudal to the vestibular nuclei. The amplification of some responses following removal of descending inputs from the vestibular nuclei is consistent with the known descending projection from the medial vestibular nucleus to the InM (Carleton and Carpenter 1983).
